# An unusually acute abdominal pain: A case report

**DOI:** 10.1097/MD.0000000000042615

**Published:** 2025-06-13

**Authors:** Ya-Chen Wang, Jian-Feng Zhang, Hong-Yuan Shen, Chengqi Guan

**Affiliations:** a Department of Gastroenterology, Affiliated Hospital of Nantong University, Nantong, Jiangsu Province, China.

**Keywords:** acute abdominal pain, bezoar, case report, diabetes ketoacidosis, intestinal obstruction

## Abstract

**Rationale:**

Acute abdominal pain is a common clinical symptom. Its etiology is complex and intricate. This is a case of small intestinal obstruction caused by bezoar combined with diabetes ketoacidosis, which leads to abdominal pain. The obstruction was caused by bezoars, which is rare in clinical practice, and worth summarizing and learning.

**Patient concerns:**

We reported a case of a 58-year-old female who was admitted to the hospital due to “upper abdominal pain for 3 days”.

**Diagnoses:**

Fasting blood glucose was 21 mmol/L, urinary ketone body 3+, pondus hydrogenii (pH) 7.309, actual and standard bicarbonate decreased, and abdominal enhanced computed tomography showed a rounded low-density shadow with sharp edges and well-defined borders in the lumen of the bowel on the left side of the abdomen, with a size of 6 cm, and no significant enhancement within the lesion.

**Interventions:**

Including hypoglycemic treatment, correction of acid–base imbalance and electrolyte disorders, and removal of gastric stones through laparoscopy, etc.

**Outcomes:**

This abdominal pain was caused by a small intestinal obstruction caused by bezoar combined with diabetes ketoacidosis. The patient was discharged successfully and remained symptom-free during follow-up.

**Lessons:**

There are many causes of acute abdominal pain. In clinical practice, we should comprehensively consider and diagnose the causes of abdominal pain based on the patient’s current medical history, past medical history, physical examination, and auxiliary examinations. We must be alert to rare and multiple causes that can jointly cause abdominal pain, and promptly deal with different causes.

## 1. Introduction

Abdominal pain is one of the most common complaints and also one of the most common symptoms in emergency departments. There are many reasons that can cause abdominal pain, especially when it involves diseases outside the abdomen or systemic diseases. In addition, many patients have atypical symptoms, so the diagnosis of potential causes is not simple. Once misdiagnosed, it will cause huge harm to patients.

Bezoars are clumps formed by undigested substances in the stomach, which are related to factors such as diet, changes in the anatomical structure of the stomach, and poor gastric peristalsis function. The clinical manifestations are different, mild cases have no discomfort, severe cases will lead to digestive tract obstruction, digestive tract perforation, etc.

Intestinal obstruction is an important cause of acute abdominal pain. According to the etiology, intestinal obstruction can be divided into mechanical, dynamic, vascular, and unexplained intestinal obstruction. Postoperative adhesions are the most common cause of intestinal obstruction, accounting for 65% to 75%, followed by malignant tumors and intestinal hernias.^[1]^ 0.4% to 4% of mechanical gastroenteric obstruction cases are caused by bezoars.^[2]^ We are reporting a case of small intestinal obstruction caused by gastric stones, leading to abdominal pain.

Diabetes ketoacidosis is the most common acute complication of diabetes. Generally, the symptoms are obvious, but the signs are not obvious. Therefore, patients with Diabetic Ketoacidosis may also have abdominal pain, and when patients with abdominal pain are clinically encountered, we must be vigilant about whether it is caused by DKA.

The patient we reported was suffering from abdominal pain caused by intestinal obstruction combined with DKA, and the obstruction was caused by bezoars. The causes of abdominal pain and intestinal obstruction caused by these 2 causes are very rare in clinical practice, which is especially worth learning.

## 2. Case presentation

### 2.1. Chief complaints

A 58-year-old middle-aged woman who presented with upper abdominal pain and vomiting for 3 days.

### 2.2. History of present illness

The patient suddenly developed upper abdominal pain and vomiting without apparent cause. Non-projectile vomiting, vomitus is stomach contents and bile, and there is no bowel movement.

### 2.3. History of past illness

The patient has a history of hypertension, diabetes and subtotal gastrectomy. Regular oral antihypertensive and hypoglycemic drugs.

### 2.4. Personal and family history

The patient denied heart disease, or tobacco or alcohol use.

### 2.5. Physical examination

The patient’s vital signs are stable. The facial expression was painful, the abdomen was soft, and umbilical tenderness was evident, with no other positive signs.

### 2.6. Laboratory examinations

The fasting blood glucose of 21 mmol/L, urinary ketone body 3+, pH 7.309, actual bicarbonate concentration of 16.7 mmol/L.

### 2.7. Imaging examinations

**Figure 1.**
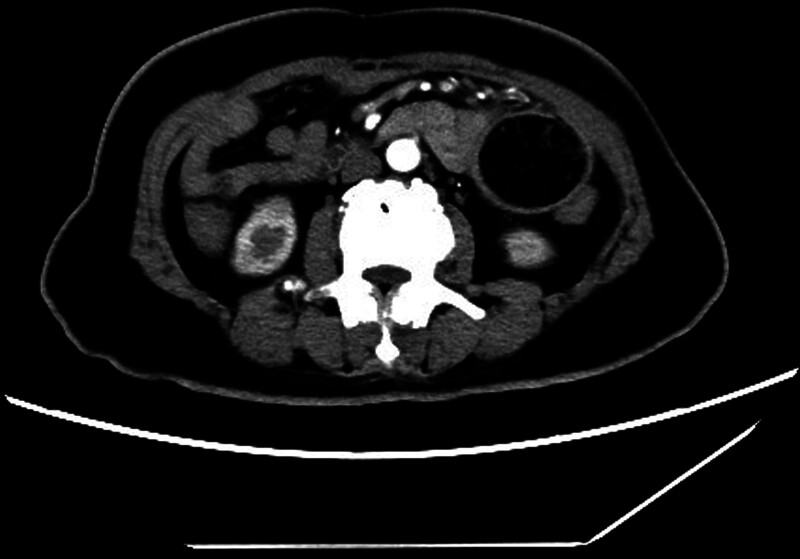
Abdominal enhanced CT: CT showed a rounded low-density shadow with sharp edges and well-defined borders in the lumen of the bowel on the left side of the abdomen, with a size of 6 cm, and no significant enhancement within the lesion. CT = computed tomography.

The gastroscopy showed residual gastritis with bile reflux. Abdominal plain CT scan showed slight dilation and fluid accumulation in the left small intestine. Abdominal enhanced CT showed a rounded low-density shadow with sharp edges and well-defined borders in the lumen of the bowel on the left side of the abdomen, with a size of 6 cm, and no significant enhancement within the lesion (Fig. 1).

## 3. Final diagnosis

The patient’s abdominal pain was caused by small bowel obstruction caused by bezoar and DKA.

## 4. Treatment

**Figure 2.**
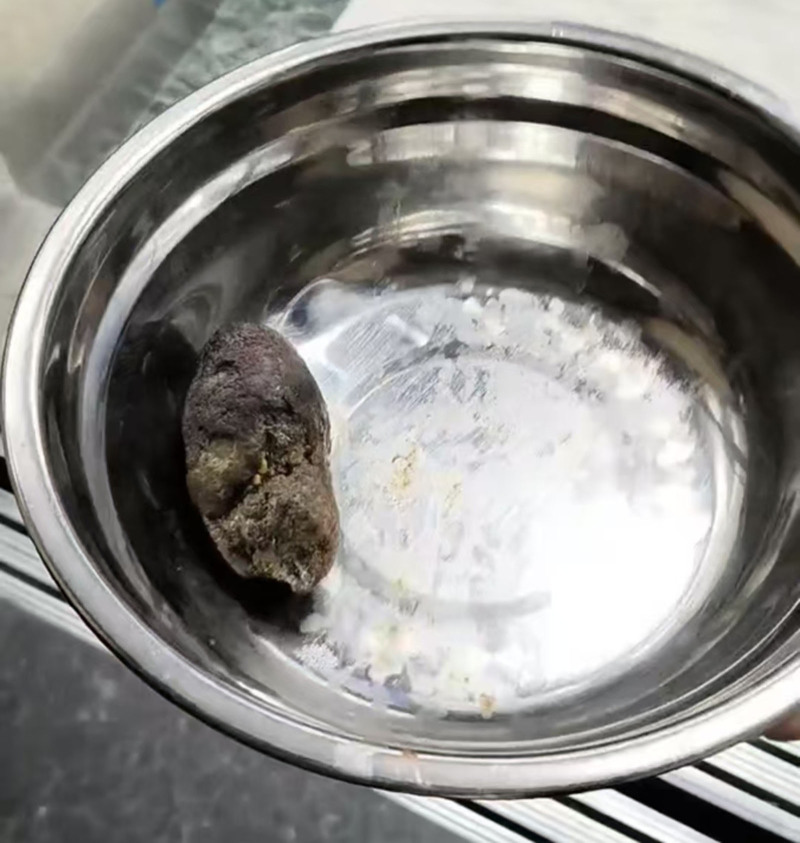
Huge, intraluminal bezoar removed from the small intestine.

The patient was treated with fluid infusion, reducing the blood sugar, correction of acid-base balance and electrolyte disorder, fasting, suppression of intestinal fluid secretion, gastrointestinal decompression, etc. After the patient’s DKA was corrected, the abdominal pain improved significantly compared to before. In addition, there was only a small amount of fluid flowing out of the gastrointestinal decompression tube. The gastrointestinal decompression tube was removed and the patient began to drink water. The patient then reappeared with abdominal pain, accompanied by vomiting. After gastrointestinal decompression, approximately 1800 mL of dark green liquid was drained out. Abdominal enhanced CT showed a bezoar in the small intestine. Due to the large size of the bezoar, and the increased frequency of abdominal pain in the patient compared to before, conservative internal medicine treatment and endoscopic treatment pose great risks. After multidisciplinary treatment, then laparoscopic exploration was performed. The distal ileum shriveled during the operation. A circular mass about 6 cm in diameter was detected, and the intestinal wall was dissected to remove the bezoar (Fig. 2). The texture of the bezoar is hard and there is no hair. Combined with the patient’s high protein diet history, it is considered that the formation of gastroliths is related to it. The patient’s abdominal pain disappeared after surgery. Discharge from hospital with improvement.

## 5. Outcome and follow-up

The patient recovered well and did not develop abdominal pain again after surgery.

## 6. Discussion

Acute abdominal pain is a common symptom in clinical practice, involving multiple organ diseases throughout the body. The etiology is complex and difficult to diagnose. Timely and accurate diagnosis of the cause of abdominal pain and reasonable treatment are very important. The most common cause of acute abdominal pain is acute gastroenteritis, followed by irritable bowel syndrome and urinary system diseases. About one-tenth of patients have acute diseases such as appendicitis, cholecystitis, pancreatitis and so on, which require immediate treatment. About one-third of abdominal pain cannot be attributed to a specific cause.^[3]^

The diagnostic approach for abdominal pain should be broad, not only considering common diseases of the digestive, cardiovascular, and respiratory systems, but also rare and uncommon diseases. For example, the 2 causes of abdominal pain in this case are not common. A comprehensive analysis based on detailed medical history, careful physical examination, and corresponding laboratory and imaging examinations is the basis for a correct diagnosis. It is also necessary to closely observe the patient’s clinical changes and response to treatment, discover valuable information from them, and timely change the diagnosis and treatment.

Bezoar refers to the lumpy material formed by indigestible substances in the stomach, and its incidence rate is 0.07% to 0.4%.^[4]^ According to the different ingested substances, they are divided into plant-based bezoars, hairy bezoars, lactic acid bezoars, and drug-induced bezoars.^[5]^ Plant-based bezoars are the most common,^[6]^ usually related to excessive consumption of foods rich in tannic acid, such as persimmons, hawthorns, etc. Under the action of stomach acid, tannic acid binds with proteins to form insoluble tannic acid proteins,^[7]^ which then adhere to plant fibers to form hard bezoars. Hairy bezoars is related to the excessive consumption of hair and other substances, and is more common in patients with mental disorders.^[8]^ Undigestible hair can inhibit gastric peristalsis, leading to more hair gathering and entangling with other substances in the stomach, forming bezoars.^[9]^ Lactic acid bezoars are formed by the coagulation of undigested milk and mucin proteins in the stomach, and are related to premature birth, delayed gastrointestinal emptying, high concentration formula milk and so on.^[10]^ Drug-induced bezoars are formed by the aggregation of different dosage forms of drugs in the stomach, which are related to the patient’s own factors (gastrointestinal stenosis, obstruction, history of gastrointestinal surgery, critical illness, etc) and drug factors (size, persistence, adhesion, etc).^[11]^

The formation of gastric stones is related to various factors. The main risk factors are changes in gastric anatomy, decreased gastric emptying function, and ingestion of indigestible substances.^[12]^ Gastric surgery changes the structure of the stomach, causing loss of pyloric function, and bilateral vagotomy significantly reduces gastric motility.^[13]^ Delayed gastric emptying caused by diabetes, mixed connective tissue disease or hypothyroidism is also a predisposing factor for the formation of gastric stones.^[14]^ In this case, the patient had a previous subtotal gastrectomy and a history of diabetes, which are all factors leading to the formation of gastric stones.

The clinical manifestations of gastric stones are not specific, and some patients do not have clinical manifestations, only incidentally discovered during examination. Some patients may experience upper abdominal pain, postprandial fullness, vomiting, etc, ^[15]^ some patients may even experience intestinal obstruction, gastritis, gastric ulcer, gastric perforation, etc.^[16]^ Therefore, patients with gastric stones are difficult to make a diagnosis before the examination is completed. In this case, the patient’s clinical manifestations were abdominal pain and intestinal obstruction.

Gastroscopy is the most commonly used method for diagnosing gastric stones, with an accuracy rate of up to 100%. However, some elderly people with poor cardiopulmonary function, severe infections, etc, who cannot tolerate gastroscopy and gastric stones entering the small intestine, need to use other examination methods. 50% to 75% of cases can be diagnosed through plain abdominal radiograph.^[17]^ Barium examination is contraindicated for patients with complete intestinal obstruction caused by gastric stones. Gastric stones appear as a mass in the lumen of the gastrointestinal tract under ultrasound, with curved surface echoes and clear posterior acoustic shadows.^[18]^ Gastric stones appear as round or oval masses at the site of obstruction on CT, with bubbles inside and mottled exterior.^[19]^

The treatment of gastric stones is mainly divided into 3 categories: medication, endoscopic treatment, and surgery. Drug therapy mainly includes acid suppressants, prokinetic drugs, various digestive enzymes, cola and so on. Endoscopic treatment is the only conservative internal medicine treatment method for patients who have failed drug therapy, mainly including endoscopic mechanical lithotripsy, laser lithotripsy, microwave lithotripsy, high-frequency current lithotripsy, electric hydraulic lithotripsy, and thermal probe lithotripsy. Surgical treatment is necessary for intestinal obstruction caused by bezoars. The surgery is usually a gastric or intestinal incision to remove bezoars. For complex patients, gastric or intestinal resection is required for treatment.^[20]^ The patient had large stones that had caused complete small bowel obstruction and ischemia, so an enterotomy was performed.

Diabetes ketoacidosis is one of the common acute complications of diabetes. Typical diabetes ketoacidosis is not difficult to diagnose, but it is easy to be misdiagnosed as acute abdomen when abdominal pain is the first manifestation. The main causes of abdominal pain in diabetic ketoacidosis are electrolyte disturbance, accumulation of acidic metabolites, hyperosmotic pressure and hypoperfusion, and increased intrabiliary pressure, etc. Generally, after replenishing fluids, lowering blood sugar, correcting acid-base and electrolyte imbalances, patients’ abdominal pain symptoms will improve. The patient had high blood glucose, high urinary ketone body, low PH, and the diagnosis of DKA was clear. After the treatment of replenishing fluids, lowering blood sugar, correcting acid-base imbalance, the abdominal pain was significantly improved.

Abdominal pain caused by intestinal obstruction due to gastric stones has a relatively low incidence rate and usually has triggers. The abdominal pain caused by diabetic ketoacidosis is generally atypical, and will disappear as blood sugar is corrected. Therefore, this compound cause of the case is very rare in clinical practice. This case report has certain enlightenment for medical workers and buys time for the further treatment of patients.

## 7. Conclusion

There are many reasons that can cause acute abdominal pain, and this report is a rare case of abdominal pain caused by bezoars leading to intestinal obstruction combined with DKA. In clinical practice, we need to comprehensively differentiate and diagnose the causes of abdominal pain in patients based on their current medical history, past medical history, physical examination, and auxiliary examinations. We should be alert to rare and multiple causes of abdominal pain, and treat patients based on different causes.

## Author contributions

**Formal analysis:** Hong-Yuan Shen.

**Writing – original draft:** Ya-Chen Wang.

**Writing – review & editing:** Chengqi Guan, Jian-Feng Zhang.
